# Optimization on condition of epigallocatechin-3-gallate (EGCG) nanoliposomes by response surface methodology and cellular uptake studies in Caco-2 cells

**DOI:** 10.1186/1556-276X-9-291

**Published:** 2014-06-10

**Authors:** Xiaobo Luo, Rongfa Guan, Xiaoqiang Chen, Miao Tao, Jieqing Ma, Jin Zhao

**Affiliations:** 1 Zhejiang Provincial Engineering Laboratory of Quality Controlling Technology and Instrumentation for Marine Food, China Jiliang University, XueYuan Road 258#, 310018 Hangzhou, People's Republic of China; 2 Hubei Collaborative Innovation Center for Industrial Fermentation, Hubei University of Technology, Lizhi Road, 430068 Wuhan, China; 3 Zhejiang Provincial Key Laboratory of Biometrology and Inspection and Quarantine, China Jiliang University, Hangzhou 310018, People's Republic of China

**Keywords:** EGCG, Nanoliposomes, Optimization, Response surface methodology, Stability, Cellular uptake

## Abstract

The major component in green tea polyphenols, epigallocatechin-3-gallate (EGCG), has been demonstrated to prevent carcinogenesis. To improve the effectiveness of EGCG, liposomes were used as a carrier in this study. Reverse-phase evaporation method besides response surface methodology is a simple, rapid, and beneficial approach for liposome preparation and optimization. The optimal preparation conditions were as follows: phosphatidylcholine-to-cholesterol ratio of 4.00, EGCG concentration of 4.88 mg/mL, Tween 80 concentration of 1.08 mg/mL, and rotary evaporation temperature of 34.51°C. Under these conditions, the experimental encapsulation efficiency and size of EGCG nanoliposomes were 85.79% ± 1.65% and 180 nm ± 4 nm, which were close with the predicted value. The malondialdehyde value and the release test *in vitro* indicated that the prepared EGCG nanoliposomes were stable and suitable for more widespread application. Furthermore, compared with free EGCG, encapsulation of EGCG enhanced its inhibitory effect on tumor cell viability at higher concentrations.

## Background

Epigallocatechin-3-gallate (EGCG) is the major and most active constituent in green tea [1]. A number of studies reported that EGCG had significant bioactivities such as anticancer [2,3], prevention of cardiovascular disease [4], and regulation of endocrine [5] and immune system [6]. EGCG has great potential in cancer prevention because of its safety, low cost, and bioavailability [7,8]. Some research results verified that encapsulated EGCG retained its bioactivity such as inducing apoptosis of Du145 prostate cancer cells.

One of the significant efforts towards these aims has been the use of colloidal delivery systems such as liposomes and micro or nanoparticles [9,10]. There have been considerable research works on the liposomes' application of protection in food and pharmacy system [11-13]. Besides, nanoliposomes have been demonstrated to possess the advantages of improving the targeting and absorption into the intestinal epithelial cells [14]. In this study, nanoliposomes could be used as potential carriers in the food system. Nanoliposomes with chemotherapeutic agents can target tumor cells either passively or actively. Passive targeting exploits the characteristic features of tumor biology that allow nanoliposomes to accumulate in the tumor by enhanced permeability and retention effect. Active targeting achieves this by conjugating nanoliposomes containing chemotherapeutics with molecules that bind to overexpressed antigens or receptors on the target cells [15]. Nanoliposomes can increase the absorption of EGCG with their ability to deliver poorly soluble drugs effectively [16]. Nanoliposomes entrap hydrophilic EGCG and use the overexpression of fenestrations in cancer neovasculature to increase EGCG concentration at tumor sites and control its release [17].

Response surface methodology (RSM) is a rapid technique used to empirically derive functional relationship between one or more than one experimental response and a set of input variables [18]. Furthermore, it may determine the optimum level of experimental factors required for the given response(s). Response surface methodology has been successfully used to model and optimize biochemical and biotechnological processes related to food [19,20]. Zhang et al. studied phosphatidylcholine proportion, cholesterol proportion, and lipids/drug ratio on preparing the nobiliside A liposome [21]. A similar trend has been reported for gypenoside liposome [22].

The main objective of this study aimed at knowing the effect of the ratio of phosphatidylcholine and cholesterol (*w*/*w*), EGCG and Tween 80 concentration (*w*/*v*) (Sigma-Aldrich, St. Louis, MO, USA), and the preparation techniques of EGCG nanoliposomes such as rotary evaporation temperature (°C) on the encapsulation efficiency and size in order to find out the optimal conditions for preparing the EGCG nanoliposomes using RSM. Nanoliposomes were tested *in vitro* for their stability in simulated gastrointestinal juice. Furthermore, EGCG nanoliposomes were used to evaluate the cellular uptake, and their effects on tumor cells were also investigated.

## Methods

### Materials

EGCG was purchased from Xiecheng Biotechnology Company (Hangzhou, China). Phosphatidylcholine (PC) and cholesterol (CH) were purchased from Beijing Shuangxuan Microorganism Co. Ltd (Beijing, China). Chloroform and diethyl ether were obtained from Hangzhou Jiachen Chemical Company (Hangzhou, China). All other chemicals were of reagent grade. The water used for all experiments was distilled twice through an all-glass apparatus.

### Preparation of EGCG nanoliposomes

EGCG nanoliposomes were prepared by reverse-phase evaporation method [23,24]. Briefly, a certain amount of PC and CH was dissolved in chloroform-diethyl ether, and EGCG was dissolved in a phosphate-buffered solution (PBS; 0.20 M, pH 7.4). The organic phase was mixed with the aqueous phase by probe sonication for 5 min. The mixture was placed in a round-bottom flask, and a gel was formed by evaporating the organic solvent under reduced pressure using a rotary evaporator. Then, 30-mL phosphate-buffered solution containing Tween 80 was added and evaporated for another 20 min.

### Encapsulation efficiency determination

The encapsulation efficiency (EE) of EGCG nanoliposomes was calculated to determine the concentration of entrapped EGCG in nanoliposome and unentrapped EGCG in the aqueous phase. Respectively, the EGCG nanoliposomes were separated from the aqueous phase using a freeze centrifuge (GL 20A, Sorvall Biofuge Stratos Co., Fisher Scientific, Leicestershire, England). A 0.5-mL liposome suspension was taken and spun at 13,000 rpm for 30 min at 4°C. The same suspension was ruptured using sufficient volume of ethanol, and the total amount of EGCG was determined spectrophotometrically.

The percentage of encapsulating efficiency (EE%) was calculated according to Equation 1 [25].

(1) $$\mathrm{EE}\%=\frac{W_{2}-W_{1}}{W_{2}}\times 100,$$

where *W*_1_ is the amount of free EGCG, and *W*_2_ is the total amount of EGCG present in 0.5 mL of nanoliposomes.

### Particle size

The mean vesicle size of the nanoliposomes was measured by a laser scattering method (Nano ZS 90, Malvern, UK). Liposomal suspensions were diluted 100-fold with double-distilled water before the measurement. The determination was repeated three times per sample for three samples.

### Experimental design and optimization

RSM as a generic method for optimization was applied to optimize the formulation of EGCG nanoliposomes. The optimization was designed based on a four-factor Box-Behnken design with a total of 27 experimental runs. Based on the preliminary experiments and our previous studies, four formulation parameters which included PC/CH ratio (*X*_1_), EGCG concentration (*X*_2_), Tween 80 concentration (*X*_3_), and rotary evaporation temperature (*X*_4_) were identified as key factors responsible for the EE and size. In view of the feasibility of liposome preparation, the ranges of the four factors were determined as follows: PC/CH (3 to 5, *w*/*w*), EGCG concentration (4 to 6, *w*/*v*), Tween 80 concentration (0.5 to 1.5, *w*/*v*), and rotary evaporation temperature (30°C to 40°C) (Table 1). The response could be related to the selected variables by a second-order polynomial model. In this study, a second-order polynomial (Equation 2) was used to generate response surfaces.

**Table 1:** Independent variables and their levels in the experimental design

**Independent variables**	**Symbols**	**Code levels**		
		**-1**	**0**	**1**
PC/CH (*w*/*w*)	*X*_1_	3	4	5
EGCG concentration (*w*/*v*)	*X*_2_	4	5	6
Tween 80 concentration (*w*/*v*)	*X*_3_	0.5	1	1.5
Rotary evaporation temperature (°C)	*X*_4_	30	35	40

(2) $${\hat{Y}}_{i}=\beta_{0}+\sum_{i}\beta_{i}X_{i}+\sum_{i}\beta_{\mathrm{ii}}{X_{i}}^{2}+\sum_{i\neq j}\beta_{\mathrm{ij}}X_{i}X_{j},$$

where ${\hat{Y}}_{i}$ represents the predicted responses, *X*_*i*_ and *X*_*j*_ are the coded values of independent variables, *β*_0_ is the intercept coefficient, *β*_*i*_ are the linear coefficients, *β*_*ii*_ are the squared coefficients, and *β*_ij_ are the interaction coefficients [26]. Statistical significance of the terms in the regression equations was examined. The significant terms in the model were found by analysis of variance (ANOVA) for each response. The adequacy of the model was checked accounting for *R*^2^ and adjusted *R*^2^. The desired goals for each variable and response were chosen. All the independent variables were kept within the range while the responses were either maximized or minimized.

### Malondialdehyde value

EGCG nanoliposomes were stored in a refrigerator at 4°C for 30 days. The malondialdehyde (MDA) value was determined as an index of the phospholipid peroxidation [27]. The MDA value was detected spectrophotometrically by thiobarbituric acid (TBA) reaction following the method of Weng and Chen [28]. Taking 5 mL of a mixture of 25 mmol/L TBA, 0.9 mol/L TCA and 50 mmol/L HCl in a test tube and 1 mL EGCG nanoliposomes were heated to 100°C for 30 min, and after reaching room temperature, the absorbance of the solutions was measured at 532 nm [29].

### *In vitro* release of EGCG from nanoliposomes

The controlled release was examined in simulated gastric juice of pH 1.3 and intestinal juice of pH 7.5. The solution of pH 1.3 consisted of HCl (0.10 M), pepsin, and deionized water, while the solution of pH 7.5 was made up of KH_2_PO_4_ (6.8 mg/mL), NaOH (0.10 M, adjusted to pH 7.5), trypsin (10 mg/mL), and deionized water [30]. Five milliliters of EGCG nanoliposome suspensions was mixed with the equal volume of simulated gastrointestinal juice in a 50-mL beaker. The beaker was placed on a magnetic stirrer adjusted to a constant speed of 150 rpm at 37°C. Aliquots of 0.2 mL were sampled from the beaker at predetermined intervals. The release of EGCG from nanoliposomes was evaluated by a release ratio. The release ratio was calculated using Equation 3 [31].

(3) $$\mathrm{Release}\;\mathrm{ratio}\%=\left(1-\frac{{\mathrm{EE}}_{t}}{{\mathrm{EE}}_{0}}\right)\times 100,$$

where EE_0_ is the encapsulation efficiency of EGCG nanoliposomes before incubation, and EE_*t*_ is the encapsulation efficiency of EGCG nanoliposomes after incubation for the time.

### Cellular uptake studies

Cell viability was determined by methyl thiazolyl tetrazolium (MTT) reduction assay [32,33]. Caco-2 cells (CBCAS, Shanghai, China) were cultured in DMEM (Gibco, Gaithersburg, MD, USA). The cells were cultured at 37°C with 5% CO_2_[34]. The cells were passaged thrice a week. At 80% confluence, the cells were subcultured into 96-well plates. After the monolayer of cells became formed for 36 h, the cells were treated with a range of concentrations of different EGCG nanoliposomes and EGCG. The cells were treated with the described particle suspensions for 24 h. Cell activity was determined by measuring the enzymatic reduction of yellow tetrazolium MTT to a purple formazan, as measured at 570 nm using an enzyme-labeled instrument [35].

### Statistical analysis

The results were expressed as mean ± standard deviation of three independent experiments. Values of *p* > 0.05, *p* < 0.05, and *p* < 0.01 were considered not significant, significant, and extremely significant, respectively. SPSS 16.0 software was used for the statistical analysis.

## Results and discussion

### Fitting the model

For the corresponding fitting of the explanatory models, the variations of encapsulation efficiency and size were analyzed. These analyses indicated that adding terms up to quadratic significantly improved the model (Table 1) and could be the most appropriate model for the response variable.

Regression analysis and the analysis of variance (ANOVA) were used for fitting the model and to examine the statistical significance of the terms. The estimated regression coefficients for the response variable, along with the corresponding *R*^2^, adjusted R^2^ (adj-*R*^2^), *F* value, and *p* value of lack of fit, were shown in Table 2.

**Table 2:** ANOVA and regression coefficients of the second-order polynomial model for the response variables (actual values)

**Source**	**DF**	**EE (%)**			**Size (nm)**		
		**Coefficient**	**Sum of squares**	***p *** **value**	**Coefficient**	**Sum of squares**	***p *** **value**
Model	14	84.31	5,214.51	<0.0001	182.33	17,393.67	<0.0001
Linear							
*X*_1_	1	-3.44	142.35	0.0166	0.58	4.08	0.7894
*X*_2_	1	-5.18	321.99	0.0013	6.42	494.08	0.0110
*X*_3_	1	5.25	331.07	0.0011	-5.08	310.08	0.0348
*X*_4_	1	-2.36	66.55	0.0815	-5.25	330.75	0.0302
Quadratic							
*X*_1_^2^		-12.21	794.46	<0.0001	-34.87	6,486.75	<0.0001
*X*_2_^2^		-17.80	1,689.58	<0.0001	2.63	36.75	0.4286
*X*_3_^2^		-15.91	1,350.02	<0.0001	-22.88	2,790.75	<0.0001
*X*_4_^2^		-13.91	1,031.75	<0.0001	-17.88	1,704.08	0.0001
Interaction							
*X*_1_*X*_2_		-9.68	374.81	0.0007	-8.50	289.00	0.0404
X_1_*X*_3_		17.60	1,238.34	<0.0001	-6.00	144.00	0.1308
*X*_1_*X*_4_		4.45	79.30	0.0601	26.25	2,756.25	<0.0001
*X*_2_*X*_3_		5.17	106.81	0.0330	-9.25	342.25	0.0279
*X*_2_*X*_4_		-0.17	0.12	0.9372	24.50	2,401.00	<0.0001
*X*_3_*X*_4_		-2.56	26.11	0.2567	15.00	900.00	0.0016
Residual	12		220.91			657.00	
Lack of fit	10		214.09	0.1452		628.33	0.1999
Pure error	2		6.82			28.67	
Total	26		5,435.42			18,050.67	
*R*^2^		0.9594			0.9636		
Adj-*R*^2^		0.9119			0.9211		
CV		7.43			4.94		

The lack of fit showed that the models failed to represent the data in the experimental domain at which points were not included in the regression. The lack of fit of the EE and size were 0.15 and 0.20, respectively, which were not significant (*p* > 0.05) for the response surface model, meaning that the model represented the data accurately.

The *R*^2^ values for the response variable of the EE and size were both 0.96 which were higher than 0.80, indicating that the regression models were suitable to explain the behavior, but a large value of *R*^2^ does not always imply the adequacy of the model. Adding a variable to the model will always increase *R*^2^, regardless of whether the additional variable is statistically significant or not. Thus, it is better to use an adj-*R*^2^ to evaluate the model adequacy. The *R*^2^ and adj-*R*^2^ values for the model did not differ greatly, indicating that nonsignificant terms have not been included in the model.

### Encapsulation efficiency

The *p* values were used as a tool to check the significance of every coefficient. The smaller the magnitude of *p* is, the more significant the corresponding coefficient is. Values of *p* less than 0.05 indicate that model terms are significant.

**Figure 1:**
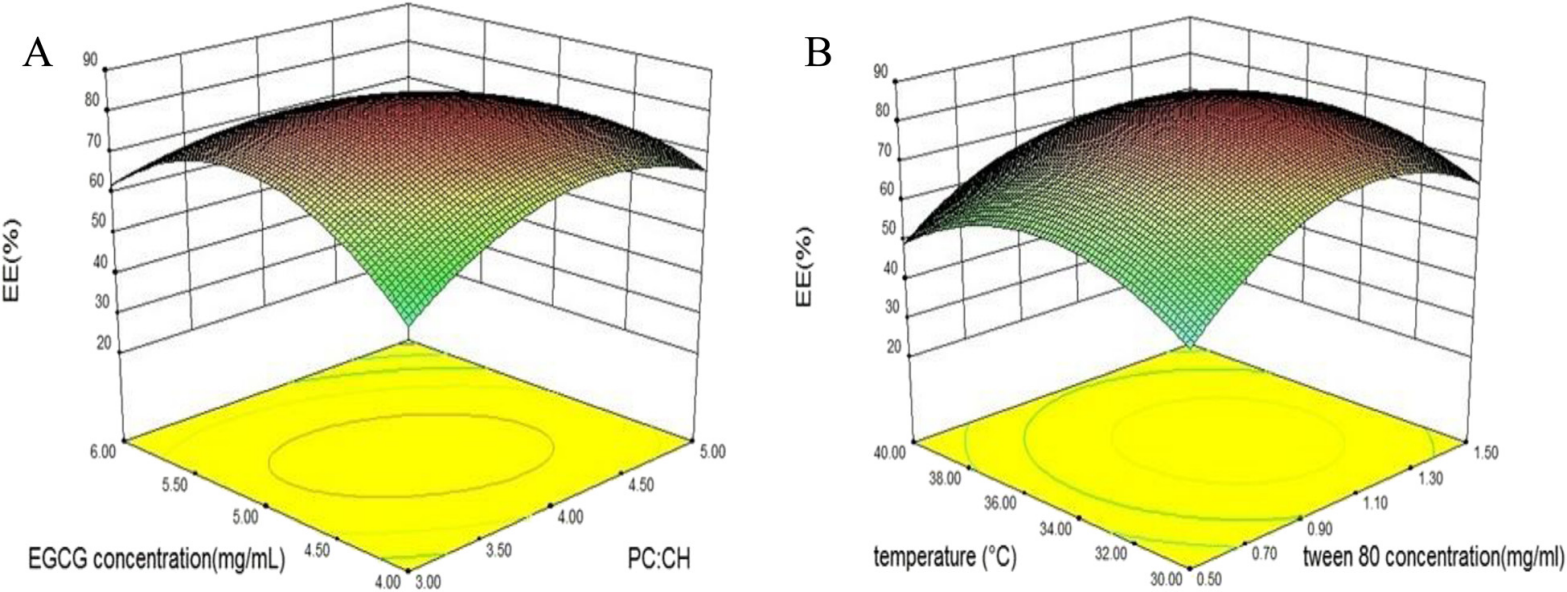
**Response surface for the effects of independent variables on encapsulation efficiency of EGCG nanoliposomes.** The effects of phosphatidylcholine-to-cholesterol ratio and EGCG concentration were shown in **(A)** (rotary evaporation temperature = 35°C and Tween 80 concentration = 1 mg/mL); the effects of rotary evaporation temperature and Tween 80 concentration were shown in **(B)** (phosphatidylcholine-to-cholesterol ratio = 4 and EGCG concentration = 5 mg/mL).

The results in Table 2 showed that the linear effects of phosphatidylcholine-to-cholesterol ratio, EGCG concentration, and Tween 80 concentration were significant (*p* < 0.05), whereas rotary evaporation temperature was not significant. The effects of the independent variables on EGCG nanoliposomes were shown in Figure 1. According to Figure 1A, increasing the phosphatidylcholine-to-cholesterol ratio increased the encapsulation efficiency. It might be due to the fact that cholesterol can change the order of mobility of lecithin in the lipid bilayer, thus reinforcing the membrane stability. On the other hand, increasing the EGCG concentration increased the encapsulation efficiency. At higher EGCG concentration, the encapsulation efficiency was enhanced because more EGCG was encapsulated into the vesicles.

As shown in Figure 1B, the increase in Tween 80 concentration led to the increase in the EE of EGCG nanoliposomes. This increased EE may be attributed to the increase in densification of liposome surface due to the availability of lipophilic ambience, which could accommodate EGCG to a higher extent [36]. The results indicated the higher level of phosphatidylcholine-to-cholesterol ratio and EGCG and Tween 80 concentrations increased the encapsulation efficiency.

### Particle size

The *p* values were used as a tool to check the significance of every coefficient. The smaller the magnitude of *p* is, the more significant the corresponding coefficient is. Values of *p* less than 0.05 indicate that model terms are significant.

**Figure 2:**
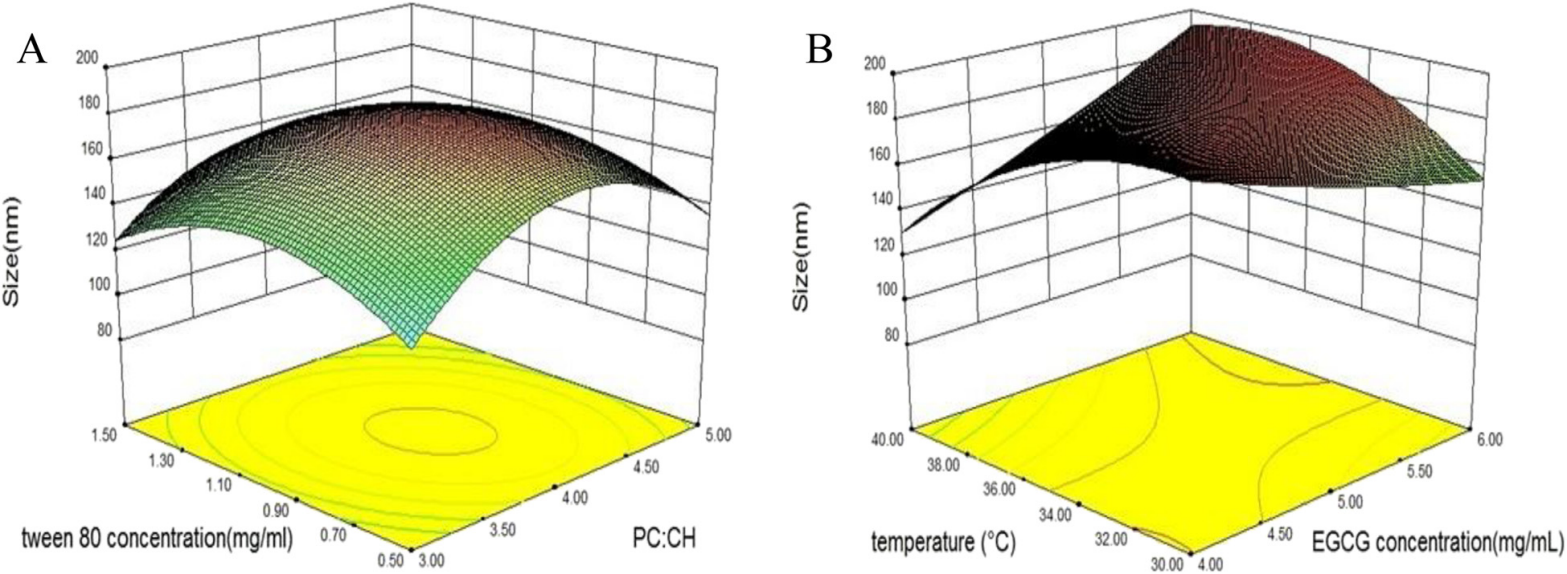
**Response surface for the effects of independent variables on the size of EGCG nanoliposomes.** The effects of phosphatidylcholine-to-cholesterol ratio and Tween 80 concentration were shown in **(A)** (EGCG concentration = 5 mg/mL and rotary evaporation temperature = 35°C); the effects of EGCG concentration and rotary evaporation temperature were shown in **(B)** (phosphatidylcholine-to-cholesterol ratio = 4 and Tween 80 concentration = 1 mg/mL).

The results in Table 2 showed that based on the sum of squares, the importance of the independent variables on yield could be ranked in the following order: EGCG concentration > rotary evaporation temperature > Tween 80 concentration > phosphatidylcholine-to-cholesterol ratio.The variation of size with the phosphatidylcholine-to-cholesterol ratio and Tween 80 concentration is presented in Figure 2A. The particle size of the EGCG nanoliposomes decreased with decreasing phosphatidylcholine concentration because phospholipids constitute the liposome membrane and phosphatidylcholine concentration directly affected the particle size of the liposome.

The effect of the EGCG concentration and rotary evaporation temperature on the nanoliposome size is given in Figure 2B. The rotary evaporation temperature had an effect on the size of the liposomes. Zhou et al. reported that during the preparation, the lipid solution temperatures are critical parameters for the character of the gemcitabine liposome injection [37]. Besides, it has also been cited that different EGCG concentrations have an effect on the particle size and dispersion of the liposome. Similar trend has been reported for paclitaxel magnetic nanoparticle liposome [38].

### Optimization

**Figure 3:**
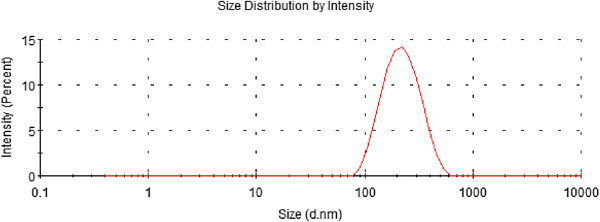
The particle size of the optimized EGCG nanoliposomes.

After the effects of PC/CH, EGCG concentration, Tween 80 concentration, and rotary evaporation temperature on the formulation of EGCG nanoliposomes were investigated, the optimum ranges for each independent variable were found to generate EGCG nanoliposomes with the highest EE and small size. The optimum formulation conditions were as follows (Table 3): phosphatidylcholine-to-cholesterol ratio of 4.00, EGCG concentration of 4.88 mg/mL, Tween 80 concentration of 1.08 mg/mL, and rotary evaporation temperature of 34.51°C. The conditions gave the highest encapsulation efficiency (85.79% ± 1.65%) with the low value of the particle size (180 nm ± 4 nm), and the experimental values were close to the predicted values (Table 4), which indicated that the optimized preparation conditions were very reliable. EGCG nanoliposomes of optimized formulation were used for the determination of particle size distribution (Figure 3). The results indicated that the model used can identify operating conditions for preparing EGCG nanoliposomes.

**Table 3:** Predicted optimum conditions for the preparation of EGCG nanoliposomes

**Factor**	**Low**	**High**	**Optimum**
Phosphatidylcholine/cholesterol	3	5	4
EGCG concentration (mg/mL)	4	6	4.88
Tween 80 concentration (mg/mL)	0.5	1.5	1.08
Rotary evaporation temperature (°C)	30	40	34.51

**Table 4:** Predicted and experimental values of the responses obtained at optimum conditions

**Response**	**Predicted value**	**Experimental value**
EE (%)	85.14	85.79 ± 1.65
Size (nm)	181	180 ± 4

Results are shown as the mean ± SD (*n* = 3).

### Malondialdehyde value

**Figure 4:**
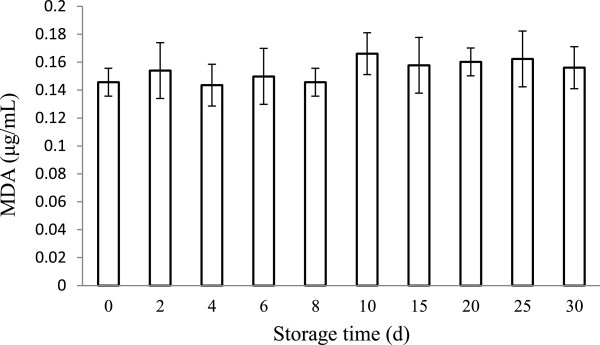
**Variation of the MDA values in EGCG nanoliposomes during storage at 4°C for 30 days.** Data reported are the mean values ± standard variation of three replications.

Phospholipid was used as the major component of liposomal membrane, containing partially polyunsaturated fatty acid residues sensitive to oxidative free radicals [39]. The MDA, which is a final product of fatty acid peroxidation, was evaluated in the study. During 30 days of storage at 4°C, the MDA values in the EGCG nanoliposomes showed no distinct differences (*p* > 0.05) in the MDA values which were shown in Figure 4. The result showed the EGCG nanoliposomes could be stable in a period of time in fatty acid peroxidation field. Similar results were observed in some studies [40]. Additionally, to consummate stability research, storage stability, effect of sonication, and other aspects which also evaluate the stability of the nanoliposomes with respect to variations in their pH and leakage rates are ongoing.

### *In vitro* release of EGCG from nanoliposomes

**Figure 5:**
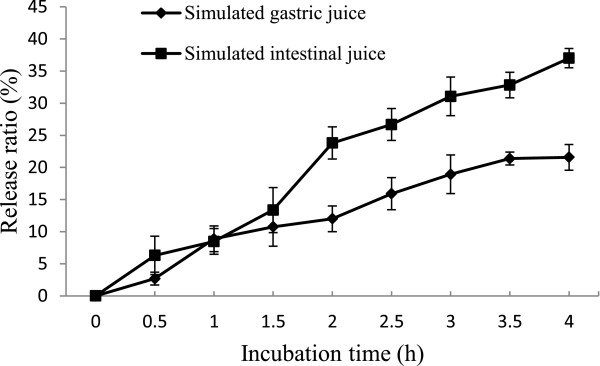
**The effect of simulated gastrointestinal juice on EGCG nanoliposomes.** Data reported are the mean values ± standard variation of three replications.

When EGCG nanoliposomes could be used as carriers for the oral administration of EGCG, they must be able to withstand passage through the stomach and small intestine. *In vitro* release has been used as a very important surrogate indicator of *in vivo* performance. Guan et al. have found that 23% and about 37% of lactoferrin released from nanoliposomes in the simulated gastric/intestinal juice were considered to be stable [40]. *In vitro* release profiles of EGCG from nanoliposomes were shown in Figure 5. About 21% EGCG was released from nanoliposomes within 4 h in the simulated gastric juice. The instability of the nanoliposomes would be related to the permeation of protons, and the release of EGCG from nanoliposomes in the simulated gastric juice may be due to the low pH [41]. However, because food usually remains in the stomach for more or less 4 h, the liposomal EGCG could be effectively protected in the gastric juice. In simulated intestinal juice, bile salts and pancreatic lipase may cause the EGCG release from nanoliposomes [42]. This effect may increase the release of nanoliposome. The nanoliposomes showed an acceptable stability and may be fit for use in the oral administration [43]. Previous studies suggested that many liposome compositions used were unstable in the conditions prevailing in the gastrointestinal tract through *in vitro* tests [44,45]. It has been demonstrated that liposomes were pinocytosed by intestinal epithelial cells and transferred to the serosal side of the gut by means of more stable liposomes in an everted gut system [46]. Our study on EGCG nanoliposomes has shown that there may be the possibility of enhancing the uptake process to deliver a range of drugs by the oral route. In future research, particle sizes which affect absorption efficiency in the stomach and intestine should be determined as an index of the stability of nanoliposomes.

### Cell viability

**Figure 6:**
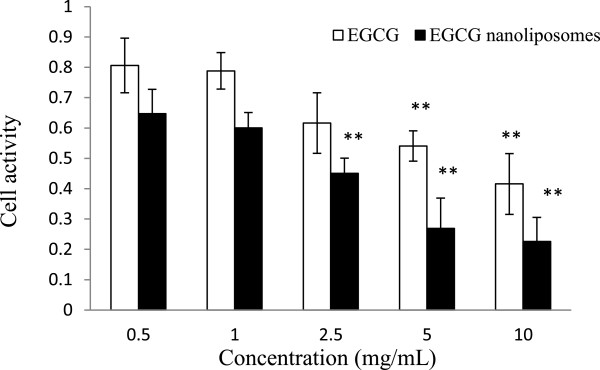
**Cell viability of Caco-2 cells treated with different concentrations of EGCG nanoliposomes and EGCG.** Data reported are the mean values ± standard variation of three replications. (** *p* < 0.01, compared with the first group).

After the cells were incubated with 0.5, 1, 2.5, 5, and 10 mg/mL of EGCG nanoliposomes for 24 h, they were compared with the control experiments. Figure 6 showed that the MTT results demonstrated a concentration-dependent uptake after exposure to EGCG nanoliposomes. With the same concentration, the cell activity of the EGCG nanoliposomes was lower than the cell activity of EGCG. IC_50_s of EGCG and EGCG nanoliposomes were 6.13 and 1.47 mg/mL, respectively. The MTT results showed that EGCG nanoliposomes and EGCG activated in the cells in a manner of dose-effect relation and EGCG nanoliposomes had a more obvious function to the tumor cells (*p* < 0.01) without affecting normal cell viability. The possibility of both targeting drugs to specific tissues and cells and facilitating their uptake and cytoplasmic delivery had rendered liposomes a versatile drug carrier system with numerous potential applications [47], which were expected to increase the efficiency and effectiveness of the drug as well as enable the use of new (and more potent) drugs [48]. In the latter application, reducing the particle size of nanoliposomes may be an efficient and reliable tool for improving the bioavailability and absorption in food and medicine [49].

## Conclusions

The effects of the phosphatidylcholine-to-cholesterol ratio, concentration of EGCG and Tween 80, and rotary evaporation temperature on preparing EGCG nanoliposomes were studied. A second-order polynomial model was obtained for predicting the encapsulation efficiency and size. Increasing the phosphatidylcholine-to-cholesterol ratio, EGCG concentration, and Tween 80 concentration increased the encapsulation efficiency. Numerical optimization determined the optimum preparation conditions, which were the phosphatidylcholine-to-cholesterol ratio of 4.00, EGCG concentration of 4.88 mg/mL, Tween 80 concentration of 1.08 mg/mL, and rotary evaporation temperature of 34.51°C. Under these conditions, the experimental encapsulation efficiency and size of the EGCG nanoliposomes were 85.79% ± 1.65% and 180 nm ± 4 nm, which were close with the predicted value. Therefore, the optimized preparation conditions were very reliable. The value of MDA indicated the stability of the EGCG nanoliposomes suspension. Furthermore, nanoliposomes were tested *in vitro* for their stability in simulated gastrointestinal juice. The results indicated that the prepared EGCG nanoliposomes were stable and may be fit for use in the oral administration. The cellular uptake of the EGCG nanoliposome formulations were found to depend on the concentration. In conclusion, we have demonstrated that EGCG nanoliposomes with different concentrations could modulate the growth of tumor cells and were suitable for more widespread application.

## Abbreviations

Caco-2 cells: human epithelial colorectal adenocarcinoma cells; CH: cholesterol; CV: coefficient of variation; DF: degree of freedom; DMEM: Dulbecco's modified eagle medium; EE: encapsulation efficiency; EGCG: epigallocatechin-3-gallate; MDA: malondialdehyde; MTT: methyl thiazolyl tetrazolium; PC: phosphatidylcholine; RSM: response surface methodology; SD: standard deviation; TBA: thiobarbituric acid; TCA: tricarboxylic acid.

## Competing interests

The authors declare that they have no competing interests.

## Authors' contributions

XL came up with the idea, contributed to the design of the experiment, and agreed with the paper's publication. RG and MT conducted most of the research experiments and drafted the manuscript. JM and XC analyzed the data, drew the pictures, and refined the research thesis. XC and JZ revised the manuscript critically. All authors read and approved the final manuscript.

## Authors' information

XL and RG shared first authorship, their institution and address is shown as follows:

Zhejiang Provincial Engineering Laboratory of Quality Controlling Technology and Instrumentation for Marine Food, China Jiliang University, XueYuan Road 258#, Hangzhou 310018, People's Republic of China.
